# Reverse Shoulder Arthroplasty for Acute Proximal Humerus Fractures Treated With Trabecular Metal Prosthesis: Medium-Term Results

**DOI:** 10.7759/cureus.34652

**Published:** 2023-02-05

**Authors:** Shashidharan Viswanathan, Anjali Hema Kashyap, Harish Kashyap Shanker

**Affiliations:** 1 Trauma and Orthopedics, Royal Alexandra Hospital, NHS Greater Glasgow and Clyde, Paisley, GBR; 2 Medical Sciences and Nutrition, University of Aberdeen, Foresterhill Health Campus, Aberdeen, GBR

**Keywords:** tuberosity healing, reverse total shoulder replacement, reverse shoulder arthoplasty, shoulder fracture dislocation, proximal humeral fracture

## Abstract

Introduction

Reverse shoulder arthroplasty (RSA) is becoming increasingly popular as a primary procedure for complex proximal humeral fractures (PHF) in acute trauma due to more emerging evidence and better patient outcomes.

Methods

This study is a retrospective case series of 51 patients who underwent a trabecular metal RSA for non-reconstructable, acute three or four-part PHF performed by a single surgeon between 2013 and 2019 with a minimum follow-up of three years. This included 44 females and seven males. Mean age was 76 years (range: 61-91 years). Oxford shoulder score (OSS) along with relevant patient information relating to demographics and functional outcomes were collected at regular intervals in outpatient clinic follow-ups. Complications were addressed accordingly during treatment and follow-up.

Results

The mean follow-up duration was 5.08 years. Two patients were lost to follow-up and nine patients died due to other causes. Four of them had developed severe dementia and were excluded as an outcome score from them could not be acquired. Two patients who had surgery beyond four weeks post-injury were excluded. Thirty-four patients in total were followed up. Patients had good range of motion and mean OSS of 40.28 post-operatively. The overall complication rate was 11.7%, and none of the patients had deep infections, scapular notching, or acromial fractures. Revision rate was 5.8% at mean follow-up of five years and one month (range: three years to nine years two months). Greater tuberosity union following intra-operative repair was evident on radiographs in 61.7% of the patients.

Conclusion

RSA is certainly a rewarding surgery in patients with complex PHF and was associated with good post-operative OSS along with patient satisfaction, and positive radiological outcomes at minimum three-year follow-up.

## Introduction

The prevalence of proximal humerus fractures (PHF) accounts for around 4-10% of all fractures as reported in several studies conducted in different populations [1]. These fractures are common in elderly patients and treatment can be challenging, especially when they are displaced and un-reconstructible.

Most of the minimally displaced fractures are managed conservatively. Displaced three or four-part fractures, particularly in elderly patients, present with challenges. Reconstruction of these fractures with poor bone quality is associated with avascular necrosis and a high incidence of implant failure. Hemiarthroplasty is an option in these patients, but functional results are unpredictable; this is largely due to a deficiency of tuberosity healing including migration, resorption, and malunion [2]. The emergence of reverse shoulder arthroplasty (RSA) has been able to provide better function though not always equivalent to preinjury levels in these patients [3]. Our study aimed to evaluate the outcomes of the use of trabecular metal RSA with a minimum three-year follow-up performed by a single surgeon.

Biomechanically, the glenohumeral joint of the shoulder is a multiaxial ball and socket articulation between the humeral head, and a shallow pear-shaped bony glenoid to permit a wide range of motion [4]. The shallow glenoid is deepened by the circumferential glenoid labrum that along with the capsule and ligaments form the static stabilizers. The glenoid is titled upward about five degrees and retroverted about five degrees from the scapular body axis. The humeral head is retroverted about 20 degrees in relation to the distal humerus inter-epicondylar axis [5]. The humeral neck-shaft angle of 130-140 degrees creates a shearing force at the glenohumeral joint during shoulder movements. The rotator cuff muscles (supraspinatus, infraspinatus, teres minor, and subscapularis) arising from the scapular body and inserted into greater and lesser tuberosities provide a compressive force and act as dynamic stabilizers during glenohumeral motion.

While in reverse shoulder arthroplasty, the anatomy is reversed to a glenoid ball (glenosphere) and concave proximal humerus component [6]. The design of reverse shoulder replacement helps to transform shear forces into compressive forces creating a rotational moment even when part or all the rotator cuff muscles are non-functional. The deltoid muscle initiates abduction of the shoulder with the non-functional rotator cuff. In the RSA, the center of rotation of the shoulder is displaced inferiorly and medially. This also lengthens the moment arm and increases the deltoid force [7].

## Materials and methods

This was a retrospective case series (Level of Evidence IV). Fifty-one consecutive Trabecular metal shoulder replacements following trauma from 2013 to 2019 were enrolled in the study. This study was exempt from the National Health Service (NHS) Research Ethics Committee review with Integrated Research Application System (IRAS) project ID #323730.

**Figure 1:**
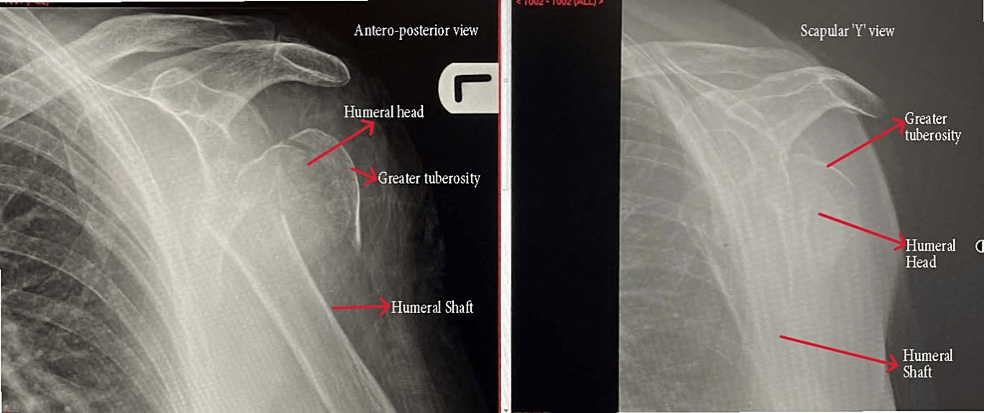
Comminuted fracture proximal humerus. Un-reconstructible proximal humerus fracture (PHF) on antero-posterior and scapular "Y" view. Fractured fragments (parts) are labeled in the diagram.

Inclusion criteria were un-reconstructible, acute three or four-part proximal humerus fractures (PHF) with or without dislocation (Figure 1). Patients with other associated injuries, lower limb fractures, were excluded from the study. Fifty-three percent (n=27) of the fractures were associated with dislocation. Ten patients had three-part fractures and 14 patients had four-part fractures. Exclusion criteria included alcohol or other substance abuse, dementia, neurological disease, or associated glenoid fractures.

Patients in the age range of 61-91 years were included in the study, with the mean age being 76 years. This figure included 44 females (86%) and seven males (14%). Majority of these elderly patients had one or more major medical co-morbidities including diabetes mellitus, hypertension, ischemic heart disease, or chronic kidney disease. Eighteen percent (n=9) of the patients were either previous or current smokers.

Nine patients died from other conditions during follow-up. Two patients were lost to follow-up. Four of them had developed severe dementia and were excluded as an outcome score from them could not be acquired. Two patients who had surgery beyond four weeks post-injury were excluded. The reasons for the delay in operation were due to patients taking longer time to decide on treatment options and the lack of theatre slots available on trauma lists. We considered injuries less than four weeks old as acute since there was little scarring found during dissection and hence no effect on results. The study included a total of 34 patients.

All operations were performed in beach chair position reclined at 30 degrees with support under the scapula, with a mechanical arm holder (AssistArm surgical positioner; Utica, NY: CONMED Corp.) under general anesthetic and regional nerve blocks. Deltopectoral approach was used. Long head of biceps (LHB) and rotator interval were identified. LHB was tenodesed to pectoralis major. Greater and lesser tuberosities with cuff attachments were identified and debulked, but bony attachments were retained to promote tuberosity healing and held with a stay suture. Comminuted and devitalized humeral head fragments were removed. We used the Zimmer Biomet Trabecular metal reverse shoulder system (Warsaw, IN: Zimmer, Inc.).

The glenoid was prepared first, with the arm held in external rotation at the shoulder. Trabecular metal baseplate was positioned over the glenoid with inferior translation and tilt to achieve least rocking forces during shoulder movements [8]. It was impacted to the glenoid and held securely with two additional screws, which were locked to the base plate after insertion of locking caps. The glenospheres are available in two following sizes: 36 mm and 40 mm in diameter. The glenosphere would attach to the Morse taper on the base plate.

**Figure 2:**
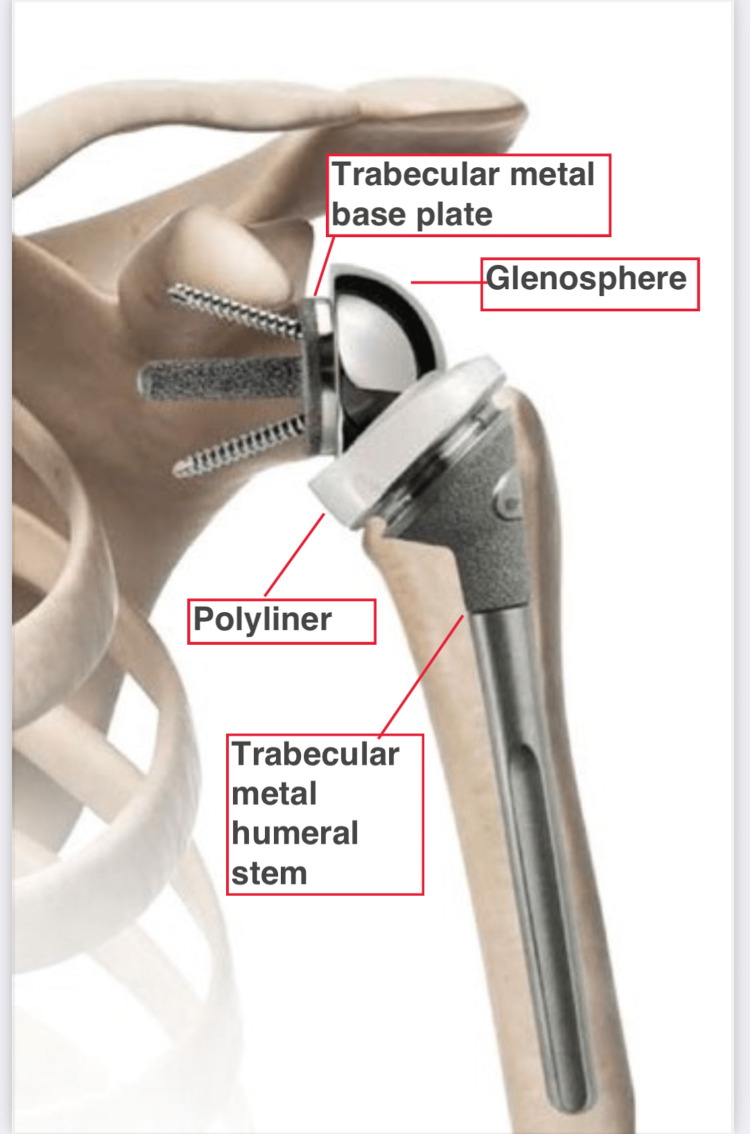
Trabecular metal reverse shoulder replacement. Proximal part of the implant stem has a trabecular metal coating that came in contact with the tuberosities to promote bony ingrowth. The image was provided by Zimmer Biomet with written permission.

Trabecular metal reverse humeral stem was used (Figure 2). This is a low-profile inlay stem, with a neck shaft angle of 143 degrees. It has six holes in the metaphyseal area to attach the tuberosities. This is not a fracture-specific stem and could be used uncemented in elective cases. However, in fractures due to proximal comminution and bone loss, cemented fixation was preferred without pressurization over an intra-medullary cement restrictor. Stem was placed in 20 degrees of retroversion in relation to the forearm.

The proximal part of the stem had a trabecular metal coating that came in contact with the tuberosities to promote bony ingrowth. In cases with severe comminution, insertion of pectoralis major was used as a guide to achieve the correct height and tension. Two drill holes were made in the shaft to reattach the tuberosities. A pair of non-absorbable looped sutures were also passed from each of the tuberosity fragments onto the holes in the proximal part of the stem. Tuberosities were approximated to the shaft first with a "nice sliding knot" and then tied together.

The ease of approximation was an indication of adequate height and correct polyethylene insert size for the humeral component. Patients were placed in a polysling for four weeks, seen by the physiotherapist on the ward, and followed up in the community. In the first four weeks after the operation, patients were allowed to start with active range of motion exercises for elbow and hand and passive forward elevation of shoulder. Polysling was discarded at four weeks and active assisted range of motion was commenced up until the sixth post-operative week. Active range of motion of the shoulder was started after week six.

**Figure 3:**
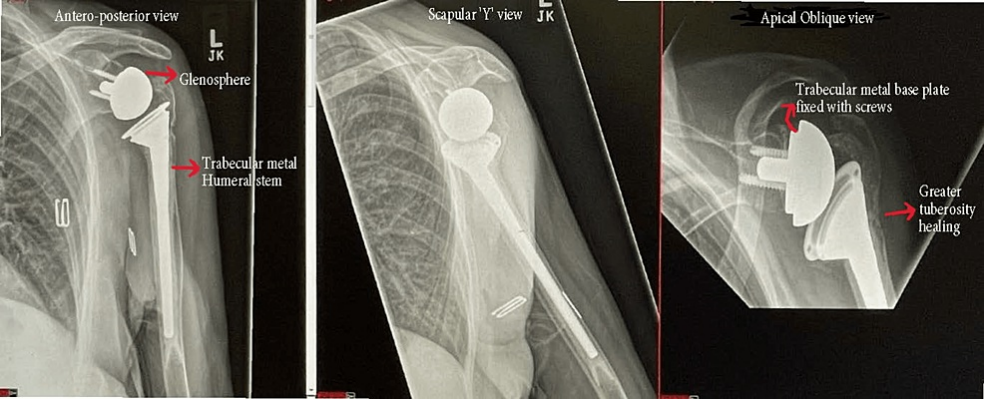
Reverse shoulder arthroplasty (RSA) during follow-up with healed greater tuberosity. Antero-posterior, scapular "Y" and apical oblique views at post-operative follow-up showed healed greater tuberosity and well-fixed implants.

All patients were followed up in the clinic at three months, six months, one year, and then annually with follow-up radiographs. Oxford shoulder scores (OSSs) were collected during the follow-up visits to the clinic. We have used the last scoring obtained from the patient to assess outcome at the longest follow-up period post-operatively. All patients had shoulder antero-posterior view, scapular "Y" view, and an apical oblique or axial view (Figure 3). Tuberosity healing was assessed, and they were deemed to have healed when the gap was less than 5 mm with no further displacement from initial positioning on radiographs [9]. Complications were assessed by radiographic and clinical records.

## Results

The mean age was 76 years (range: 61-91 years of age). The majority of patients in the cohort were females 86% (n=44) with 14% males (n=7). Fifty-five percent of injuries were left-sided (n=28), and 45% of injuries were right-sided (n=23). The final analysis included 34 patients. Seventeen patients were excluded from this study. The time from the date of injury to operation ranged from one to 28 days, with a mean of 10.5 days. Length of hospital stays ranged from one to 28 days, with a mean of five days. The procedure was carried out by a single surgeon at a single center. Mean follow-up duration was five years and one month (range: three years to nine years two months).

Range of motion was assessed clinically, and Oxford shoulder scores (OSSs) were obtained at final follow-up (Table 1). Mean OSS was 40.28 (range: 25-48) post-operatively. Forward elevation and abduction at final follow-up were recorded in the notes in 94% of patients. Mean forward elevation and mean abduction were 114 degrees (range: 45-160 degrees) and 96 degrees (range: 40-140 degrees), respectively. External rotation was documented in 87% of patients with a mean of 30 degrees (range: 15-60 degrees). Internal rotations were recorded in 90% of the patients followed up to three years. Fourty-eight percent of these patients could reach mid-spine level. Greater tuberosity union following intra-operative repair was evident on radiographs in 61.7% of the patients.

**Table 1:** Active range of motion.

Active range of motion	Recorded in % of patients	Mean (range) in degrees
Forward elevation	94%	114° (range: 45-160°)
Abduction	94%	96° (range: 40-140°)
External rotation	87%	30° (range: 15-60°)
Internal rotation	90%	48% could reach mid-spine level

Early complications of superficial infection, urinary tract or respiratory tract infections, acute delirium or acute kidney injury were prevalent in seven (20.5%) patients (Table 2). Early complications listed here are within the first 30 days during the post-operative period. These patients recovered well and were discharged.

**Table 2:** Early post-operative complications encountered within the first 30 days.

Early complications	Frequency
Deep implant infection	0
Superficial infection	1
Respiratory tract infection	2
Urinary tract infection	2
Acute delirium	1
Post-operative acute kidney injury	1

Four patients (11.7%) developed late complications which included aseptic loosening, peri-prosthetic fracture, or dislocation (Table 3). Complications encountered 30 days after the operation were listed as late complications. None of the patients had deep infections needing further procedures. Aseptic loosening was noticed in a patient who presented with loosening of stem three years after the operation. Two staged revision was done. There was no microbiological evidence of infection from the intra-operative tissue samples.

**Table 3:** Late post-operative complications encountered during the follow-up.

Late complications	Frequency
Deep implant infection	0
Superficial infection	0
Aseptic loosening	1
Peri-prosthetic fracture	1
Acromial stress fractures	0
Dislocation	2

One patient with dislocation had initially presented four weeks post-injury with axillary nerve palsy. A second patient dislocated their shoulder five weeks after the operation following a fall and trauma to the operated shoulder. Both dislocated shoulders were revised with a new liner but redislocated again after six weeks. Both patients refused further intervention due to the risks involved with multiple surgeries.

One patient had a peri-prosthetic fracture distal to the tip of stem (Wright and Cofield type C) following a fall on the operated side eight months after surgery [10]. The patient initially underwent conservative management in a humeral brace but this failed to achieve union of the fracture. This was fixed at six weeks with a neutralization plate and screws and went on to healing.

The overall revision rate was 5.8%. We did not encounter any scapular notching, acromial fractures, or base plate failures in our patient series. Thirty patients (88%) said they thought this operation was a success and would recommend it to other patients.

## Discussion

Treatment of displaced three or four-part fractures has always been challenging [11]. Even with the advent of locking plates, securing adequate purchase in osteopenic humeral head to load the weight of the arm is challenging. This is due to the quality of bone being poor and osteopenic in the age and sex group who commonly sustain these injuries. Although hemiarthroplasty is an option, it relies entirely on tuberosity healing [2,12].

**Figure 4:**
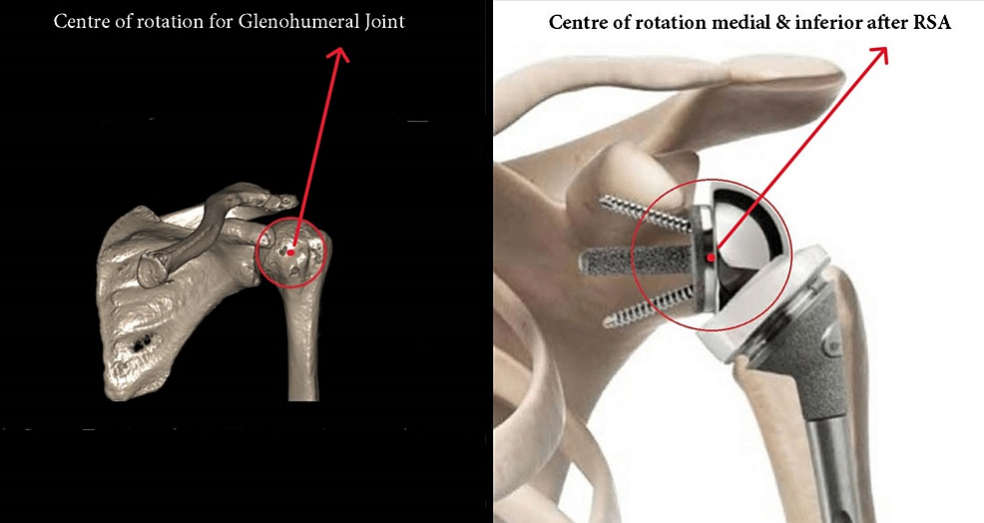
Biomechanics comparing normal glenohumeral joint to reverse shoulder arthroplasty (RSA). The images show that the center of rotation of glenohumeral joint is medial and inferior after reverse shoulder arthroplasty and the deltoid muscle is lengthened and tensioned. The image was provided by Zimmer Biomet with written permission.

Reverse shoulder arthroplasty (RSA) has gained popularity in all parts of the world as a treatment option for these fractures as they do not rely on tuberosity fixation and healing. The success of reverse shoulder arthroplasty is biomechanically based on increasing the moment arm of the deltoid muscle. This is possible as the center of rotation of the glenohumeral joint is moved medially and inferiorly (Figure 4). This also reduces the forces applied on glenoid and baseplate interface during shoulder motion.

Our study was from a case series of 51 patients from a single center. All cases were under a single surgeon and included a minimum of three years of follow-up, with an average period of follow-up being five years and one month. The range of motion achieved by patients, tuberosity healing, and complications in the long-term follow-up were comparable with other studies reported in the literature (Table 4).

**Table 4:** Comparison with other studies. *Standard deviation (SD). **American Shoulder and Elbow Surgeons (ASES) score (0-100). ***Constant score (CS) (0-100). ^****^Oxford Shoulder score (OSS) (0-48).

Studies	Number of patients	Total follow-up (in months)	Active Forward elevation in degrees	Active Abduction in degrees	Active External rotation in degrees	Functional scores	Tuberosity healing (% of patients)	Complications
Cuff et al. [9]	24	30 (range: 24-48)	139° (range: 102°-172°)	-	24° (range: 8°-42°)	ASES^**^ 77 (range: 67-82)	83%	-
Bufquin et al. [13]	43	22 (range: 6-58)	97° (range: 35°-160°)	86° (range: 35°-150°)	8° (range: -40°-40°)	CS^***^ 44 (range: 16-69)	41.5%	27.9%
Dezfuli et al. [14]	13	34	119°	-	27°	ASES^**^ 82	-	-
Villodre- Jimenez et al. [15]	30	34.5 (SD^*^=19.3)	124° (SD^*^=30.3°)	95° (SD^*^=34.7°)	13° (SD^*^=28°)	CS*** 49.1 (SD^*^=14.1)	-	13.3%
Klein et al. [16]	20	33.3 (range: 24-52)	123° (range: 60°-175°)	113° (range: 60°-180°)	25° (range: 10°-35°)	CS^***^ 67.9 (range: 47-98)	-	-
Wright et al. [17]	21	56±25	130°±31°	-	32°±18°	ASES^**^ 82±13.5	70%	14.3%
Barbosa et al. [18]	35	38.3	132° (range: 70°-160°)	109° (range: 70°-140°)	38° (range: 0°-70°)	CS^***^ 64.4 (range: 38-85)	85.7%	12.8%
Current study	34	61 (range: 36-110)	114° (range: 45°-160°)	96° (range: 40°-140°)	30° (range:15°-60°)	OSS^****^ 40.28 (range: 25-48)	61.7%	11.7%

Bufquin et al. in a retrospective case series assessed the clinical outcome of reverse shoulder arthroplasty (RSA) in 43 elderly patients over 65 years of age with comminuted proximal humerus fractures (PHF) [13]. They discussed that the main difficulty encountered with reverse shoulder arthroplasty was the fixation of the tuberosities in an anatomical position and this was the main prognostic factor in influencing the functional outcome. They reported 12 complications including neurovascular injury, dislocation, intra-operative glenoid fracture, and scapular notching.

Dezfuli et al. in their case series of 49 patients compared RSA for acute PHF, malunion or non-union, failed PHF hemiarthroplasty, and failed PHF fixation [14]. They concluded that RSA is an effective treatment option for PHF as both a primary and a revision procedure.

Villodre-Jiménez et al. found that RSA is a valid option to treat three and four-part proximal humerus fractures (PHF) in elderly patients [15]. The surgical goals should include the anatomical reconstruction of the tuberosities. Klein et al. had good functional results even without tuberosity repair for RSA done through juxta-acromial approach for RSA in elderly patients with PHFs [16].

Wright et al. from their study with a 56-month follow-up of uncemented stems in RSA said that cement may not be necessary for the trauma setting [17]. Cuff et al. reported in their case series of 53 patients that reverse shoulder arthroplasty resulted in better clinical outcomes and a similar complication rate compared with hemiarthroplasty for the treatment of comminuted PHF in the elderly at 30-month follow-up [9].

Barbosa et al. at a minimum two-year follow-up concluded that there were no significant differences in outcomes between patients with healed and reabsorbed tuberosities [18]. Jain et al. in a systematic review and meta-analysis concluded that during RSA, repairing the tuberosities give better clinical outcomes in terms of patient satisfaction and range of motion [19]. Chun et al. from their study of patients in two groups did not note significant differences in functional outcomes and range of motion, except for external rotation, in the anatomically healed tuberosity group [20].

Limitations of our study are that this is a case series and only 67% of patients were followed up to three years. More prospective long-term studies will better highlight the benefit of this increasingly popular procedure for treatment of proximal humerus fractures (PHF) in elderly patients.

## Conclusions

The results of the current study are good and similar in comparison to other studies. Un-reconstructible proximal humerus injury can be debilitating if left untreated, and so reverse shoulder arthroplasty is certainly a rewarding surgery. Improved implant designs including modularity and advanced surgical techniques have continued to provide good outcomes in elderly low-demand patients. Through further long-term prospective studies, longevity of reverse shoulder arthroplasty in younger patients, implant survival rate, mode of failure of components, and revision techniques are to be explored in the future.
